# Scientific activity by medical students: the relationship between academic publishing during medical school and publication careers after graduation

**DOI:** 10.1007/s40037-019-0524-3

**Published:** 2019-07-09

**Authors:** Cathelijn J. F. Waaijer, Belinda W. C. Ommering, Lambertus J. van der Wurff, Thed N. van Leeuwen, Friedo W. Dekker, NVMO Special Interest Group on Scientific Education

**Affiliations:** 1 Center for Innovation in Medical Education, Leiden University Medical Center, Leiden, The Netherlands; 2 Centre for Science and Technology Studies, Faculty of Social and Behavioural Sciences, Leiden University, Leiden, The Netherlands; 3 Department of Clinical Epidemiology, Leiden University Medical Center, Leiden, The Netherlands; 4 The Netherlands Association for Medical Education, Utrecht, The Netherlands

**Keywords:** Research in medical education, Medical students, Clinician-scientists, Bibliometrics

## Abstract

**Introduction:**

Engagement of clinicians in research is important for the integration of science and clinical practice. However, at this moment, there is a shortage of clinician-scientists. Success experiences can stimulate student interest in a research career. Conducting actual research leading to publication is a potential method to gain success experience. This study assessed whether publication as a medical student is associated with publication after graduation. We determined whether medical students in the Netherlands who are involved in research, as measured by publication in international journals before graduation: 1) are more likely to publish, 2) publish a greater number of papers, and 3) have higher citation impact scores after graduation.

**Methods:**

We matched 2005–2008 MD graduates (with rare names, *n* = 4145 in total) from all eight Dutch university medical centres to their publications indexed in the Web of Science and published between 6 years before and 6 years after graduation. For sensitivity analysis we performed both automatic assignment on the whole group and manual assignment on a 10% random sample.

**Results:**

Students who had published before graduation: 1) were 1.9 times as likely to publish, 2) published more papers, and 3) had a slightly higher citation impact after graduation.

**Discussion:**

Medical students who conducted research leading to a publication before graduation were more likely to be scientifically active after graduation. While this is not a causal relationship *per se*, these results cautiously suggest that successful early involvement in research could influence the long-term scientific activity of clinicians.

## What this paper adds

Scientific education is an important element in all medical curricula in the Netherlands, as it trains medical students to use research in their clinical practice and prepares a subgroup to conduct research themselves. Previous studies have shown that quite a few medical students publish a paper before graduation. However, the long-term impact of early publication on the later scientific publication career was not known. Using validated bibliometric methods, we found that publication before graduation is associated with an increased likelihood of publication after graduation, a greater number of publications after graduation and a slightly higher citation impact after graduation.

## Introduction

What’s learnt in the cradle lasts to the tomb: a saying that applies to activities like riding a bicycle. But does it also apply to the involvement of clinicians in science? All clinicians should at least be able to use research in their clinical practice, a competency required by the Netherlands Federation of University Medical Centres, the U.S. Accreditation Council for Graduate Medical Education (ACGME) and the Canadian Medical Education Direction for Specialists (CanMEDS), among others [1–3]. In addition, we need clinicians who conduct research themselves: clinician-scientists. However, there is a shortage of clinician-scientists, which is visible in multiple places in the world, for example in the United States, Canada, and Europe [4–8].

This shortage is thought to lead to undesirable effects. For example, it has been argued that clinical practice and science have become too disengaged—into patient care on the one hand and basic research on the other [9]. As a result, medical research might lose clinical relevance, while clinical problems might remain unanswered. The question is how to stimulate clinicians to become and stay involved in research. An answer may lie in scientific education during medical training [10].

Formal scientific education can take various shapes and forms [11]. These may be categorized according to student involvement: students as audience or participants. In the forms where students are the audience, learning is quite passive. However, in the forms where students are participants, students learn actively about research, which has been asserted to be a much more effective form of scientific education [12].

The ultimate form of active learning in scientific education, it can be argued, is participation of students in the scientific process. Often, this takes the form of research projects, which usually take place in the graduate phase, but may also take place in the undergraduate phase [13–15]. Undergraduate students are motivated to do research already early in their studies. This provides an opportunity to engage them in research early on in medical training [10]. The question is what the long-term outcomes are of such early engagement in research [4].

Here, we study whether publication during medical training, capped by authorship of one or more scientific publications, is associated with the post-graduation scientific activity of medical graduates. Are medical students who experience success in the sense that they successfully go through both the research process and the scientific publication process more likely to stay involved in research and keep publishing after graduation? There have been other studies that predict research engagement after medical training, but these often focus on either scholarly concentration or MD/PhD programs, not on the larger group of MD graduates, e.g., [16–18]. In addition, many of these do not directly evaluate scientific publication as an outcome variable but rather the intention to be involved in research [18–20].

We use bibliometric methods to study the relationship between pre-graduation and post-graduation publication. Bibliometric methods are especially suitable to study this relationship, as they can be used to track the scientific performance of individuals, reinforcing its strength by grouping the scores of individuals to larger sets of publications, with more robust bibliometric scores of citation impact as a result.

Specifically, we aim to study the following questions: are medical students who publish before graduation: 1) more likely to publish after graduation, 2) do they publish a greater number of papers after graduation, and 3) do they publish papers with a higher citation impact after graduation? If the answers to these questions are positive, authentic research learning opportunities during medical training and the opportunity to publish scientific work could impact students’ interest in a research career.

## Methods

All 2005–2008 MD graduates from all eight Dutch university medical centres were included in the study. All eight agreed to participate and provided the names of their graduates. With 1658 graduates in 2005, 1832 graduates in 2006, 1990 graduates in 2007, and 2064 graduates in 2008 this study includes 7544 medical graduates. The study was approved by the Educational Institutional Review Board of Leiden University Medical Center (reference number OEC/ER7RC/20171212/1) on 12 December 2017.

In the Netherlands, in 2005–2008, medical school comprised 6 years of study, of which 4 years were pre-clinical and 2 years were clinical training. Students typically start medical school directly after finishing secondary school, which means that the majority of students are approximately 18–19 years old when starting medical school and they have not previously obtained an undergraduate degree [21]. Partly because of the nature of the medical school system, MD/PhD programs in the Anglo-Saxon tradition are virtually absent. Such programs do exist but typically only draw less than twenty medical students. When medical students pursue a PhD degree, they usually do so after MD graduation (either full-time or in combination with postgraduate medical specialty training). All eight medical schools provide scientific training in line with the national Blueprint for Medical Education [3], including a compulsory full-time individual research project of at least 14 weeks in pre-clinical training.

The names of the MD graduates were matched to their publications indexed in the Centre for Science and Technologies Studies in-house version of the Web of Science database (database version complete up until week 13 of 2017). A common problem in such matches is the false-positive assignment of papers (papers that were not written by a person but still attributed to them) and false-negative assignment (papers not attributed to a person that *were* written by them). A false-positive assignment mainly results from homonyms: names shared by multiple persons. Especially in the case of common names and few initials, there is a considerable chance that a publication was not authored by the graduate in question. False negatives can occur due to spelling errors, missing initials, and changing names related to marriage or divorce. To prevent false positives and negatives, one could manually try to check all publication assignments. However, this was not feasible in our case. Our study includes 7544 graduates, of which a considerable number were expected to have published many papers after graduation.

Therefore, we employed two complementary strategies. We automatically assigned publications to a subset of all graduates with relatively rare names, a strategy also employed in other studies [22, 23]. Additionally, we manually assigned publications to a 10% random sample from this group. We selected rare names based on the number of initials and the prevalence of the last name in the Web of Science (the number of unique combinations of last name and initials). We selected all graduates with three or more initials and a last name occurring in less than 1000 unique combinations of last names and initials, and with two initials and a last name occurring in fewer than 50 unique combinations. This resulted in a set of 4145 (out of 7544) MD graduates. In addition, we used an author clustering algorithm developed by the Centre for Science and Technologies Studies [24]. The algorithm sorts all publications in the Web of Science into clusters of publications presumed to be authored by the same person. We matched the graduates’ full names (last name plus all initials) to the most common full name in an author cluster. This decreases the chance of false-positive assignment, as all initials have to match. To further decrease this chance, the first publication in the cluster also had to be published between 6 years before (as it is quite unlikely that a medical student would publish before starting their studies) and 6 years after graduation. From the clusters we collected all articles, reviews, and letters published between 6 years pre-graduation and 6 years post-graduation. This has the added benefit that also papers on which students did not use all their initials are collected, which decreases the chance of false-negative assignment (of course as long as they have other publications with all initials listed).

As a measure of citation impact after graduation, we used the mean normalized citation score of the papers published after graduation [25]. We counted the number of citations to each paper between the year of publication and two years afterwards. Papers were counted fully, i.e., each paper counts equally, regardless of whether it was authored by one or multiple authors. The citation score was then normalized by scientific field, as the number of citations that publications receive is greater in some fields than in others [25]. By definition, the normalized citation score of a field is 1; a score higher than 1.2 is considered to be above field average, a score below 0.8 lower than field average.

For statistical analyses we used SPSS Statistics version 23.0.0 (IBM). To test whether group differences were statistically significant, we used 1) the chi-square test for the likelihood to publish after graduation, 2) the Mann-Whitney U test for the number of papers published after graduation (as data were not normally distributed nor could be transformed to become normally distributed), and 3) an independent samples t‑test for the mean normalized citation impact (MNCS; Box-Cox transformed with λ = 0.75 to follow normal distribution).

## Results

### Likelihood to publish before and after MD degree

The analysis of pre- and post-graduation publication activity after automatic publication assignment showed that 518 graduates published one or more papers before or in the year of graduation (12%); 1591 graduates published after graduation (38%; Tab. 1). The relative risk of pre-graduation publication for post-graduation publication was 1.90 (χ^2^ = 185.91, 95% CI [1.76, 2.05], *p* < 0.001), which shows that MD graduates who published before graduation were almost twice as likely to publish after graduation than graduates who had not. The manual assignment of a 10% random sample of graduates (*n* = 414) with rare names showed a slightly higher number of graduates with publications. The difference lay especially in graduates who only published post-graduation. In total, manual assignment assigned publications to 32 graduates that automatic assignment did not (8%). In 27 cases, this was due to graduates publishing with fewer initials than listed in the faculty administration database, in four cases a double last name was abbreviated, and in one case the author clustering algorithm had falsely assigned a graduate’s publication to another author’s cluster. Automatic assignment did not assign any other publications than those assigned manually. Manual assignment showed 60 out of 414 graduates had published one or more papers before or in the year of graduation (14%); 192 published after graduation (46%). The relative risk was 1.60 (χ^2^ = 13.60, 95% CI [1.30, 1.98], *p* < 0.001).

**Table 1:** Number of MD graduates with publications before and after graduation (graduates with rare names only)

	**Publication after graduation** ^a^		
Publication before graduation^b^	Yes	No	Total
Yes	340	178	518
No	1251	2376	3627
Total	1591	2554	4145

^a^After graduation: between 1 and 6 years after the year of graduation

^b^Before graduation: between 6 years before or in the year of graduation

### Number of post-graduation publications

Next, we assessed whether students who published before graduation published more papers after graduation than those who did not. In total, 38% of all graduates published one or more papers after graduation. The number was heavily skewed, as of these 38%, almost a third (31%) published only one paper after graduation.

**Fig. 1:**
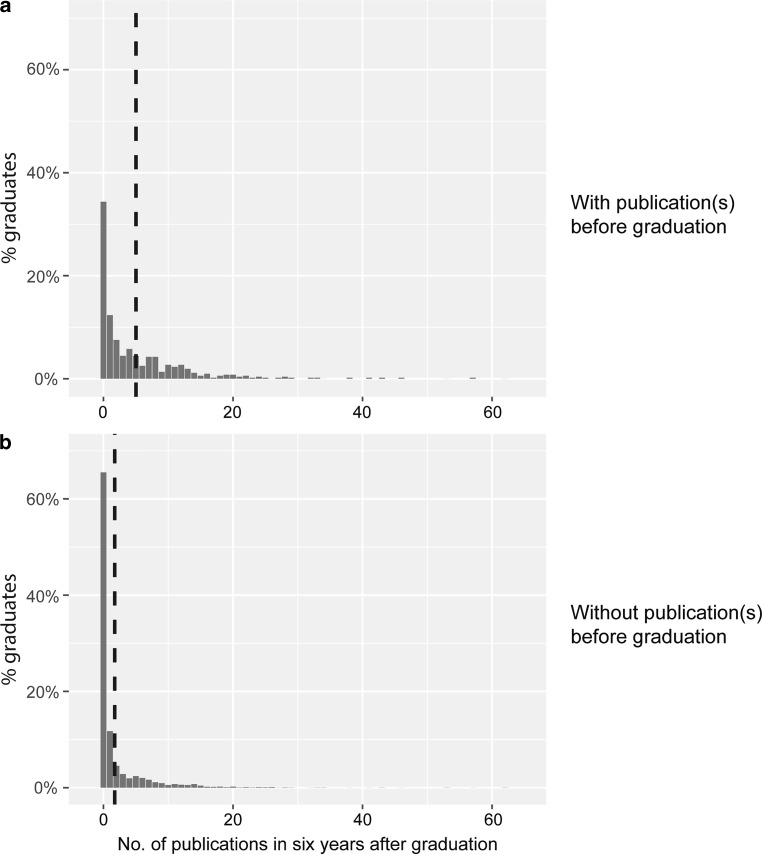
Histogram of number of publications published in 6 years after graduation by pre-graduation publication (by students with rare names). The striped line represents the mean number of publications in the 6 years after graduation for each group. Before graduation: between 6 years before or in the year of graduation

The comparison between the groups shows that for students without one or more publications before graduation, the distribution was heavily skewed to the right (Fig. 1b), whereas this distribution was less skewed for graduates with one or more pre-graduation publications using automatic assignment (Fig. 1a). The difference in the number of post-graduation publications was statistically significant (Mann-Whitney U = 1,282,058, *n1* = 518, *n2* = 3,627, *p* < 0.001 two-tailed). This is reflected in the mean number of papers published after graduation (striped line): this is 5.01 for students with pre-graduation publications (Fig. 1a, left) and 1.73 for students without pre-graduation publications (Fig. 1b, left).

The results of manual assignment again differed slightly from the results of automatic assignment. Results from manual assignment showed the mean number of publications after graduation to be 4.75 for students with pre-graduation publications (cf. 5.01 in automatic assignment) and 2.16 for students without (cf. 1.73 in automatic assignment). The distributions differed statistically significantly between the groups (Mann-Whitney U = 14,184.500, *n1* = 60, *n2* = 354, *p* < 0.001 two-tailed).

### Post-graduation citation impact

Next, we determined whether the mean citation impact of students who published before graduation differed from that of students who did not. We compared the distribution and mean of the MNCSs between students who had and had not published before graduation.

Automatic assignment showed that students who published before graduation tended to have a greater mean citation impact. The mean difference was statistically significant (*t*(1,591) = −2.81, 95% CI [−0.32, −0.06], *p* = 0.005 on Box-Cox transformed MNCS). In addition, the average of their MNCSs was higher (1.33) than that of students who did not publish before graduation (1.13). Manual assignment showed that the average MNCS of students who published before graduation was 1.12; of students who did not publish before graduation it was 1.02. This means that the MNCS of the two groups did not differ statistically significantly using manual assignment (*t*(151) = −0.61, 95% CI [−0.43, 0.22], *p* = 0.54 on Box-Cox transformed MNCS).

## Discussion

In this study, we found that medical students who published during their studies were almost twice as likely to publish after graduation, and published more papers after graduation. We also found these medical students had a slightly higher citation impact, albeit this was not statistically significant in the smaller group of manual publication assignment. This means that the early engagement of medical students in research leading to scientific publication is positively associated with sustained publication after MD graduation. Whereas this relationship may seem straightforward, no study has looked at the strength of this association before by using bibliometric methods. In addition, many studies on this topic have intended research involvement or interest in a research career as dependent variable rather than measures of actual research involvement [18–20]. It is important to note that within the studied group of medical students, all students had been required to undertake a full-time individual research project of at least 14 weeks in pre-clinical training [3]. This means that rather than looking at the effect of undertaking a research project versus not undertaking such a project, we compared students who had published before graduation, which reflects an experience of success, to those who had not. In the comparison between these groups, we found that pre-graduation publication was associated with sustained publication, a higher number of publications and higher citation impact after graduation.

Social Cognitive Career Theory, and especially its key concept of self-efficacy, could explain why such a positive association exists [26]. Mastery of an activity leads to higher self-efficacy [27]. Early involvement in research leading to the publication of a student’s scientific work could increase research self-efficacy [20, 28], which could be an explanation of our results. The effect of a success experience during medical school is not the only possible explanation of the association we found, though, as the effect of self-efficacy is not limited to the period of medical training. Career interests already develop during childhood and adolescence [26]. Certain medical students could thus have developed a greater interest in research than others already before starting medical training [20]. If these students publish more often before and after graduation, it is a confounder of the association we found between pre-graduation and post-graduation publication.

Other explanations of the association we found are the extrinsic motivation to conduct research and selection effects. A previous study by our group showed that medical students have a high extrinsic motivation to do research, already in their first year. They expect it to improve their chances for their preferred residency spot [10]. A selection effect is at play if PhD advisors prefer to hire the recent MD graduates who have published during their studies as PhD candidates; this could also contribute to the association we discovered.

On a more general level, our results show that quite a number of medical students in the Netherlands published one or more papers in the 6 years after graduation: 1591 out of 4145, which is 38%. This finding seems to disprove the clinician-scientist shortage often reported upon [4–8], and which we mentioned in the introduction. At the same time, we also noted in our Results section that the distribution of the number of publications is heavily skewed. Of the 38% who published after publication, almost one-third (31%) published only one paper. These graduates do not appear to have remained active clinician-scientists after graduation. In addition, the selection system for medical specialty residencies may have increased the number of graduates with post-graduation publications. As mentioned above, medical students are quite extrinsically motivated to pursue a PhD degree because it will increase their chances of a residency spot. It will therefore be interesting to repeat our study in a few years’ time to see how many medical graduates remain scientifically active after the period of residency spot competition has ended. Then, this basis for extrinsic motivation will have disappeared while other barriers to academic career involvement are still present, such as difficulties combining research, clinical care, and family and personal life [29, 30].

### Limitations and strengths

Naturally, our study comes with limitations, the first of which is that it only measures scientific output, both before and after graduation, due to its reliance on bibliometric methods. However, medical students and graduates may be engaged in research without that engagement leading to a publication. Case in point is the students in our studied sample who had not published before graduation. Medical school requirements in the Netherlands include a compulsory research project of at least 14 weeks [3], so these students have been involved in research but it has not led to publication.

A second limitation is that we performed an observational study and cannot infer an independent, causal effect of early scientific publication on the scientific career after graduation. For example, the aforementioned confounding effect of medical students who published before graduation possibly already having a greater interest in research than students who did not through their experiences in childhood and adolescence, may be at play [20, 26]. There is also the aforementioned selection effect of PhD advisors preferably hiring MD graduates who have published during their studies as PhD candidates. At the same time, from our results we *are* able to conclude that medical students who publish before graduation are more likely to be involved in research after graduation, publish more papers and have a slightly higher citation impact. This is regardless of whether that is because they had a greater interest in research, were more motivated, had higher research self-efficacy in the first place, were hired more often as PhD candidates, or whether the successful publication of their scientific work had a direct effect on them.

A third limitation is that the choice of bibliographic assignment (manual or automatic) affects the exact results. In a previous study by our group, we found 15% of medical students had published in the 3 years before graduation, using manual publication assignment [31]. Using manual assignment of a 10% random sample in the present study, we found a similar percentage, 14%, had published before graduation, whereas automatic assignment showed 12% of students had published in the 6 years before graduation. The discrepancy is mainly due the fact that manual assignment more easily assigns publications on which not all initials were listed.

Author clustering algorithms perform better when more information is available (including assigning publications to a cluster even when the initials do not match exactly)—this is more often the case for the prolific pre-graduation publishers who, as our study shows, publish more papers after graduation. Therefore, automatic assignment slightly underestimates the number of published papers, but more so for students who only published after graduation. Compared with manual assignment, this leads to a slight overestimation of both the relative risk of publishing after graduation by pre-graduation publication as well as a small overestimation of the difference in the number of post-graduation publications. Citation impact analysis using manual assignment *did* show material differences to automatic assignment. Not only was the average MNCS lower for all students, there was no statistically significant difference in citation impact between students with and without pre-graduation publications. However, manual assignment suffers from drawbacks, too, such as a certain subjectivity. For example, a currently active clinician-scientist often has a stronger online presence than a graduate with only one publication after graduation. In manual assignment, one would more easily assign publications to the former than the latter.

At the same time, this limitation could also be considered a strength. Although the exact results vary by choice of method, our overall conclusions of medical graduates publishing before graduation having a higher chance of publishing after graduation and publishing more papers are unaffected by the choice of method.

Another strength is that the employed bibliometric methods enabled us to study a large set of 4145 MD graduates in the Netherlands and their publications published in a 13-year period. Bibliographic assignment of publications to students is not a trivial exercise. Bierer and colleagues indicated as such in their 2015 study on the relationship between research self-efficacy and scholarship of medical students, in which they studied 248 graduates and their publications published during medical school and within 6 months after graduation [19].

## Conclusion

As mentioned in our introduction, there is currently a shortage of clinician-scientists [4–8]. Medical students who publish during their studies are more likely to keep publishing after graduation, are more productive, and have a higher citation impact. Although this association could be also caused by other factors, there is good reason to assume that the association is at least partly caused by the success experience that publication during medical school gives students [19, 20]. Medical schools could alleviate the clinician-scientist shortage by providing students with more opportunities for authentic research experiences during medical training, including the opportunity to gain experience in the scientific publication process.

In conclusion, when it comes to early scientific publication by medical students, what is learnt in the ‘cradle’ indeed lasts. Although we cannot infer from our results whether it lasts until the tomb, we do know it lasts at least during the 6‑year period after graduation.
